# Premenstrual disorders and risk of cardiovascular diseases

**DOI:** 10.1038/s44161-025-00684-4

**Published:** 2025-07-11

**Authors:** Yihui Yang, Emma Bränn, Jing Zhou, Dang Wei, Jacob Bergstedt, Fang Fang, Unnur A. Valdimarsdóttir, Elizabeth Bertone-Johnson, Donghao Lu

**Affiliations:** 1 Unit of Integrative Epidemiology, Institute of Environmental Medicine, Karolinska Institutet, Stockholm, Sweden; 2 Center for Epidemiology and Community Medicine, Region Stockholm, Stockholm, Sweden; 3 Center of Public Health Sciences, Faculty of Medicine, University of Iceland, Reykjavík, Iceland; 4 Department of Epidemiology, Harvard T.H. Chan School of Public Health, Boston, MA USA; 5 Department of Biostatistics and Epidemiology, School of Public Health and Health Sciences, University of Massachusetts Amherst, Amherst, MA USA; 6 Department of Health Promotion and Policy, School of Public Health and Health Sciences, University of Massachusetts Amherst, Amherst, MA USA

**Keywords:** Cardiovascular diseases, Public health

## Abstract

Several lines of evidence indicate a potential link between premenstrual disorders (PMDs) and cardiovascular diseases (CVDs). However, it remains unclear whether women with PMDs have a higher risk of CVDs. Here we present a Swedish nationwide population-based matched cohort study from 2001 to 2022 and a sibling matched cohort to address familial confounding. A total of 99,411 women with PMDs were included in the population analysis and 36,061 women with PMDs in the sibling analysis. Compared with individuals without PMDs, women with PMDs had a higher risk of any CVD (adjusted hazard ratio = 1.11 (95% confidence interval: 1.08–1.13) in the population analysis and 1.10 (95% confidence interval: 1.06–1.15) in the sibling analysis). The risk was particularly pronounced for PMDs diagnosed before 25 years of age and PMDs with comorbid perinatal depression. Our study shows that women who received a PMD diagnosis in specialist or primary care are at a higher risk of CVDs.

## Abstract (web summary)

Yang et al. draw evidence from population-based and sibling cohort studies to reveal that women suffering from premenstrual disorders are at an increased risk of cardiovascular disease.

## Main

Cardiovascular diseases (CVDs) are the leading cause of mortality among females^1^. Beyond CVD risk conferred by traditional risk factors, women may face an additional risk attributable to reproductive history^2^. However, current efforts did not adequately capture female-specific risk factors, and identification of more female-specific risk factors may help improve risk profiling and prediction of CVDs among females^3^.

Premenstrual disorders (PMDs), including premenstrual syndrome (PMS) and premenstrual dysphoric disorder (PMDD), constitute a constellation of emotional and somatic symptoms that cyclically emerge before the onset of menses. Around 20–30% of women of reproductive age are affected by PMS^4,5^, and 3–8% are affected by PMDD^5–8^. The mechanisms underlying PMDs are unknown, but an abnormal sensitivity to hormone fluctuation is a key mechanism^9^.

Although health outcomes of PMDs have been scarcely studied, studies have consistently found that women with PMDs are at a higher risk of vasomotor symptoms during the menopause transition^10–12^, which are important predictors of future cardiovascular risk^13^. Although causality has not been confirmed for PMDs and vasomotor symptoms, existing evidence has indicated an association between PMDs and CVDs. For example, PMDs have been associated with several risk factors for CVDs, including smoking and obesity^14,15^. In addition, studies have indicated potential common pathophysiology underlying PMDs and CVDs, including inflammation^16,17^ and dysregulation of the renin–angiotensin–aldosterone system (RAAS)^18^. However, only two studies have so far linked PMDs to CVD endpoints, with a focus on hypertension^19,20^. The long-term risk of CVDs, in addition to hypertension, is unclear in women with PMDs. Furthermore, the association between PMDs and CVDs could be confounded by factors shared within a family, for example, genetics^21,22^, and early-life exposures^23,24^, whereas no study has comprehensively addressed these confounders.

To this end, leveraging data from Swedish national registers, we aimed to examine the association between PMDs and subsequent risk of CVDs.

## Results

**Fig. 1:**
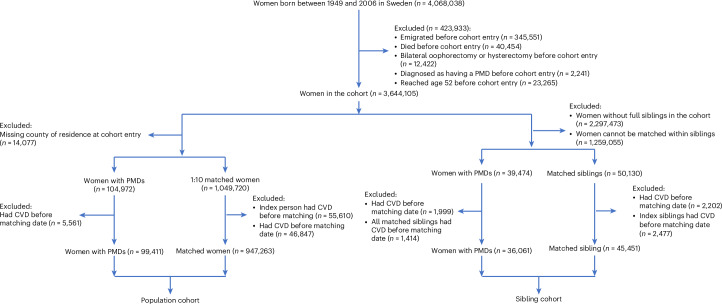
Flow chart. Among women born between 1949 and 2006, we excluded those who emigrated, died, had bilateral oophorectomy or hysterectomy, had a diagnosis of PMDs or reached age 52 years, before immigration to Sweden, age 16 years or 1 January 2001, whichever came later, leaving 3,644,105 women eligible for inclusion. Then in the population cohort, each exposed woman was matched to 10 women without PMDs at the index date, on birth year and county of residence, using incidence density sampling. In the sibling cohort, exposed individuals were matched with their sisters who were free of PMDs at the index date. In both the population and sibling cohorts, women who had CVDs before the index date were excluded, leaving 99,411 women with PMDs and 947,263 matched women in the population cohort and 36,061 women with PMDs and 45,451 unaffected sisters in the sibling cohort.

We included 99,411 women with PMDs in the populaiton cohort and 36,061 women with PMDs in the sibling cohort (Fig. 1). The median age at index date was 35.4 years in the population analysis and 34.6 years in the sibling analysis. Individuals with PMDs were more likely to have a higher educational level, higher parity and a higher prevalence of disease history (for example, psychiatric disorders and menstrual disorders) at the index date than unexposed individuals, in both the population and sibling analyses (Tables 1 and 2). In addition, in the population analysis, individuals with PMDs were more likely to be born in Sweden and use hormonal contraceptives or hormone replacement therapy (HRT) at the index date than those without PMDs.

**Table 1:** Demographic and socioeconomic characteristics of women with and without PMDs, in the population and sibling analyses

	Population analysis, *n* (%) or median (IQR)		Sibling analysis, *n* (%) or median (IQR)	
	PMDs	Non-PMDs	PMDs	Non-PMDs
Total	99,411	947,263	36,061	45,451
Age at the index date (years)	35.6 (29.3–41.5)	35.4 (29.1–41.2)	34.8 (28.8–40.5)	34.3 (27.9–41.0)
Age at the end of follow-up (years)	42.6 (35.0–50.0)	42.1 (34.6–49.7)	41.7 (34.5–49.3)	41.0 (33.5–49.6)
Birth year				
1949–1958	2,200 (2.21)	20,791 (2.19)	723 (2.00)	1,421 (3.13)
1959–1968	14,638 (14.72)	137,360 (14.50)	5,055 (14.02)	6,608 (14.54)
1969–1978	29,522 (29.70)	277,205 (29.26)	9,911 (27.48)	11,531 (25.37)
1979–1988	31,963 (32.15)	306,541 (32.36)	12,146 (33.68)	14,498 (31.90)
1989–1998	18,279 (18.39)	177,769 (18.77)	7,394 (20.50)	9,924 (21.83)
1999–2006	2,809 (2.83)	27,597 (2.91)	832 (2.31)	1,469 (3.23)
Country of birth				
Sweden	84,288 (84.79)	721,510 (76.17)	34,062 (94.46)	42,507 (93.52)
Europe	6,863 (6.90)	87,284 (9.21)	921 (2.55)	1,314 (2.89)
Other	8,257 (8.31)	138,366 (14.61)	1,078 (2.99)	1,630 (3.59)
Unknown	3 (0.00)	103 (0.01)	0 (0.00)	0 (0.00)
At the index date				
Calendar year				
2001–2007	14,747 (14.83)	143,056 (15.10)	5,586 (15.49)	7,276 (16.01)
2008–2014	29,035 (29.21)	277,005 (29.24)	10,234 (28.38)	12,846 (28.26)
2015–2022	55,629 (55.96)	527,202 (55.66)	20,241 (56.13)	25,329 (55.73)
County of residence				
Stockholm	31,872 (32.06)	302,104 (31.89)	10,605 (29.41)	11,933 (26.25)
Uppsala	4,022 (4.05)	38,379 (4.05)	1,528 (4.24)	1,884 (4.15)
Södermanland	2,373 (2.39)	22,893 (2.42)	876 (2.43)	1,165 (2.56)
Östergötland	3,729 (3.75)	36,016 (3.80)	1,401 (3.89)	1,959 (4.31)
Jönköping	2,164 (2.18)	20,997 (2.22)	931 (2.58)	1,462 (3.22)
Kronoberg	1,652 (1.66)	16,026 (1.69)	645 (1.79)	858 (1.89)
Kalmar	1,841 (1.85)	17,748 (1.87)	747 (2.07)	927 (2.04)
Gotland	804 (0.81)	7,748 (0.82)	339 (0.94)	371 (0.82)
Blekinge	1,124 (1.13)	10,775 (1.14)	432 (1.20)	605 (1.33)
Skåne	11,562 (11.63)	109,016 (11.51)	4,051 (11.23)	5,149 (11.33)
Halland	3,250 (3.27)	31,372 (3.31)	1,259 (3.49)	1,534 (3.38)
Västra Götaland	15,434 (15.53)	145,652 (15.38)	5,760 (15.97)	7,485 (16.47)
Värmland	2,443 (2.46)	23,353 (2.47)	868 (2.41)	1,131 (2.49)
Örebro	2,678 (2.69)	25,947 (2.74)	1,034 (2.87)	1,384 (3.05)
Västmanland	2,175 (2.19)	20,904 (2.21)	765 (2.12)	1,152 (2.53)
Dalarna	2,797 (2.81)	26,910 (2.84)	1,128 (3.13)	1,461 (3.21)
Gävleborg	2,236 (2.25)	21,495 (2.27)	793 (2.20)	1,098 (2.42)
Västernorrland	1,806 (1.82)	17,384 (1.84)	688 (1.91)	938 (2.06)
Jämtland	900 (0.91)	8,705 (0.92)	368 (1.02)	519 (1.14)
Västerbotten	2,719 (2.74)	26,263 (2.77)	1,101 (3.05)	1,429 (3.14)
Norrbotten	1,830 (1.84)	17,576 (1.86)	742 (2.06)	1,007 (2.22)
Educational level				
<10 years	8,592 (8.64)	103,369 (10.91)	2,551 (7.07)	4,158 (9.15)
10–12 years	38,112 (38.34)	366,982 (38.74)	14,214 (39.42)	18,619 (40.96)
≥13 years	51,935 (52.24)	456,902 (48.23)	19,155 (53.12)	22,418 (49.32)
Unknown	772 (0.78)	20,010 (2.11)	141 (0.39)	256 (0.56)
Civil status				
Cohabitated	62,840 (63.21)	578,712 (61.09)	23,239 (64.44)	29,596 (65.12)
Not cohabitated	36,568 (36.78)	367,699 (38.82)	12,820 (35.55)	15,848 (34.87)
Unknown	3 (0.00)	852 (0.09)	2 (0.01)	7 (0.02)
Income				
Quintile 1	16,580 (16.68)	196,061 (20.70)	5,538 (15.36)	7,246 (15.94)
Quintile 2	20,112 (20.23)	188,479 (19.90)	7,405 (20.53)	9,361 (20.60)
Quintile 3	20,873 (21.00)	186,886 (19.73)	7,822 (21.69)	9,691 (21.32)
Quintile 4	21,282 (21.41)	186,431 (19.68)	8,034 (22.28)	9,772 (21.50)
Quintile 5	20,561 (20.68)	188,552 (19.90)	7,260 (20.13)	9,374 (20.62)
Unknown	3 (0.00)	854 (0.09)	2 (0.01)	7 (0.02)

Continuous variables are shown as median (interquartile range), and categorical variables are shown as number (percentage).

IQR, interquartile range.

**Table 2:** Lifestyle and medical history of women with and without PMDs at the index date, in the population and sibling analyses

	Population analysis, *n* (%)		Sibling analysis, *n* (%)	
	PMDs	Non-PMDs	PMDs	Non-PMDs
Smoking^a^				
No smoking	40,464 (63.75)	356,926 (63.69)	15,000 (64.33)	17,112 (61.29)
1–9 cigarettes per day	3,974 (6.26)	30,853 (5.51)	1,385 (5.94)	1,499 (5.37)
≥10 cigarettes per day	3,469 (5.46)	31,110 (5.55)	1,213 (5.20)	1,530 (5.48)
Unknown	15,570 (24.53)	141,488 (25.25)	5,718 (24.52)	7,781 (27.87)
BMI group^a^				
Underweight	1,160 (1.83)	12,141 (2.17)	418 (1.79)	519 (1.86)
Normal	34,923 (55.02)	291,781 (52.07)	13,036 (55.91)	14,868 (53.25)
Overweight	13,854 (21.83)	124,817 (22.27)	4,976 (21.34)	5,900 (21.13)
Obese	5,195 (8.18)	58,038 (10.36)	1,894 (8.12)	2,572 (9.21)
Unknown	8,345 (13.15)	73,600 (13.13)	2,992 (12.83)	4,063 (14.55)
Parity				
0	35,934 (36.15)	386,886 (40.84)	12,745 (35.34)	17,529 (38.57)
1	15,733 (15.83)	146,392 (15.45)	5,475 (15.18)	7,112 (15.65)
2	33,838 (34.04)	277,953 (29.34)	12,552 (34.81)	13,889 (30.56)
3	10,973 (11.04)	101,387 (10.70)	4,205 (11.66)	5,364 (11.80)
≥4	2,933 (2.95)	34,645 (3.66)	1,084 (3.01)	1,557 (3.43)
Number of medications within 1 year before the index date^b^				
0	24,839 (28.32)	368,788 (44.25)	9,248 (29.29)	15,490 (39.12)
1–2	32,264 (36.79)	272,678 (32.72)	11,743 (37.19)	13,705 (34.61)
≥3	30,604 (34.89)	192,008 (23.04)	10,581 (33.51)	10,406 (26.28)
Psychiatric disorders	26,741 (26.90)	153,983 (16.26)	9,549 (26.48)	9,268 (20.39)
Initiation of hormonal contraceptives^b^	62,001 (68.32)	550,784 (63.84)	23,535 (72.05)	29,059 (70.91)
Initiation of HRT^b^	3,915 (4.31)	25,404 (2.94)	1,279 (3.92)	1,386 (3.38)
Diabetes	519 (0.52)	6,017 (0.64)	153 (0.42)	257 (0.57)
Asthma	9,907 (9.97)	70,395 (7.43)	3,553 (9.85)	3,841 (8.45)
Menstrual disorders	22,642 (22.78)	133,838 (14.13)	7,861 (21.80)	6,925 (15.24)
PCOS	2,027 (2.04)	15,488 (1.64)	737 (2.04)	836 (1.84)
Dyslipidemia	329 (0.33)	2,867 (0.30)	94 (0.26)	137 (0.30)
Autoimmune diseases	10,722 (10.79)	86,007 (9.08)	3,823 (10.60)	4,464 (9.82)

Variables are shown as number (percentage).

^a^Only in parous women. The median interval between the delivery and index year was 6 years in both the population and sibling analyses.

^b^As the Prescribed Drug Registry was incepted in July 2005, analyses on medications were limited to matching sets with the index date since 2007, and analyses on HRT and hormone contraceptives were limited to matching sets with the index date since 2006.

In the population analysis, during a median follow-up of 6.2 years (maximum 22), we identified 8,944 (12.15 per 1,000 person-years) and 73,798 (10.67 per 1,000 person-years) women with CVDs among the exposed and unexposed groups, respectively (Table 3). Compared with women without PMDs, those with PMDs had a higher risk of any CVD (hazard ratio (HR) = 1.11 (95% confidence interval (CI): 1.08–1.13) in model 3). Similar results were found in the sibling analysis (HR = 1.10 (95% CI: 1.06–1.15) in model 3).

**Table 3:** PMDs with the risk of CVDs: a nationwide register-based study in Sweden, 2001–2022

	*n*	IR	Model 1^a^		Model 2^b^		Model 3^c^	
			HR (95% CI)	*P* value	HR (95% CI)	*P* value	HR (95% CI)	*P* value
Population analysis								
Non-PMDs	73,798	10.67	Ref.		Ref.		Ref.	
PMDs	8,944	12.15	1.14 (1.11–1.16)*	<0.001	1.14 (1.12–1.17)*	<0.001	1.11 (1.08–1.13)*	<0.001
Sibling analysis								
Non-PMDs	3,540	10.64	Ref.		Ref.		Ref.	
PMDs	3,127	11.54	1.12 (1.07–1.17)*	<0.001	1.12 (1.07–1.17)*	<0.001	1.10 (1.06–1.15)*	<0.001

We used stratified Cox regression to calculate the HRs and 95% CIs, in which two-tailed Wald tests were used to calculate *P* values. We did not adjust for multiple comparison.

IR, incidence rate (per 1,000 person-years); *n*, number of cases; Ref., reference.

^a^Model 1 was adjusted for birth year and county of residence; this was performed by conditioning on matching sets in the population analysis but direct adjustment in the sibling analysis.

^b^Model 2 was additionally adjusted for educational level, country of birth, civil status, income, use of hormonal contraceptives and use of hormone therapy, all derived before the index date.

^c^Model 3 was additionally adjusted for history of psychiatric disorders before the index date.

*P < 0.05.

In the analyses of PMD subtypes, a seemingly stronger association was noted for PMDs diagnosed before age 25 years, compared with those diagnosed later, in both the population and sibling analyses (Table 4). Similar results were seen when the cutoff was set at ages 30 and 35 years (Extended Data Tables 1 and 2). In addition, a stronger association was suggested for PMDs with perinatal depression (PND) compared with those without PND, especially in the sibling analysis.

**Table 4:** Associations of subtypes of PMDs with the risk of CVDs: a nationwide register-based study in Sweden, 2001–2022

	Population analysis				Sibling analysis			
	*n*	IR	HR (95% CI)^a^	*P* value	*n*	IR	HR (95% CI)^a^	*P* value
By age at baseline								
<25 years								
Non-PMDs	2,242	3.19	Ref.		122	3.92	Ref.	
PMDs	311	4.29	1.24 (1.11–1.39)*	<0.001	119	4.53	1.41 (1.09–1.82)*	0.009
≥25 years								
Non-PMDs	71,556	11.52	Ref.		3,418	11.33	Ref.	
PMDs	8,633	13.01	1.10 (1.08–1.13)*	<0.001	3,008	12.29	1.09 (1.04–1.14)*	<0.001
By PND at baseline								
Without PND								
Non-PMDs	41,765	13.18	Ref.		2,182	13.41	Ref.	
PMDs	6,456	14.28	1.12 (1.09–1.15)*	<0.001	1,965	14.29	1.12 (1.06–1.18)*	<0.001
With PND								
Non-PMDs	2,109	8.83	Ref.		120	10.16	Ref.	
PMDs	460	12.25	1.21 (1.09–1.35)*	<0.001	141	13.78	1.68 (1.27–2.22)*	<0.001

This table shows analyses by subtypes of PMDs. In the analysis by age at baseline, individuals were grouped into PMDs <25 years and ≥25 years, with their corresponding matching population. In the analysis by PND, individuals were grouped into PMDs without and with PND, with their corresponding matching population. We used stratified Cox regression to calculate the HRs and 95% CIs, in which two-tailed Wald tests were used to calculate *P* values. We did not adjust for multiple comparison.

^a^Estimates were adjusted for birth year, county of residence, educational level, country of birth, civil status, income, use of hormonal contraceptives, use of hormone therapy and history of psychiatric disorders.

*P < 0.05.

When analyzing CVD subtypes, in both the population and sibling analyses, we found a positive association of PMDs with several subtypes of CVDs, including hypertensive diseases, essential hypertension, ischemic heart disease, cerebrovascular disease, ischemic stroke and arrhythmia (Table 5). Among all CVD subtypes, the association was seemingly most pronounced for arrhythmia in the population analysis (HR = 1.31, 95% CI: 1.25–1.37) and for ischemic stroke in the sibling analysis (HR = 1.27, 95% CI: 1.08–1.50).

**Table 5:** Associations of PMDs with the risk of subtypes of CVDs: a nationwide register-based study in Sweden, 2001–2022

		Population analysis				Sibling analysis			
		*n*	IR	HR (95% CI)^a^	*P* value	*n*	IR	HR (95% CI)^a^	*P* value
Hypertensive diseases	Non-PMDs	48,463	6.87	Ref.		2,245	6.60	Ref.	
	PMDs	5,560	7.35	1.05 (1.03–1.08)*	<0.001	1,887	6.78	1.07 (1.02–1.13)*	0.011
Essential hypertension	Non-PMDs	48,126	6.82	Ref.		2,225	6.54	Ref.	
	PMDs	5,523	7.30	1.06 (1.03–1.08)*	<0.001	1,872	6.72	1.08 (1.02–1.13)*	0.008
Other hypertensive disease	Non-PMDs	1,474	0.20	Ref.		65	0.18	Ref.	
	PMDs	177	0.22	1.08 (0.93–1.25)	0.332	61	0.21	0.93 (0.62–1.40)	0.728
Ischemic heart disease	Non-PMDs	4,573	0.63	Ref.		217	0.62	Ref.	
	PMDs	568	0.72	1.13 (1.04–1.23)*	0.005	193	0.67	1.21 (1.01–1.45)*	0.041
Cerebrovascular disease	Non-PMDs	7,021	0.96	Ref.		335	0.95	Ref.	
	PMDs	879	1.12	1.11 (1.04–1.19)*	0.002	319	1.11	1.15 (1.02–1.31)*	0.028
Subarachnoid hemorrhage	Non-PMDs	816	0.11	Ref.		37	0.10	Ref.	
	PMDs	82	0.10	0.93 (0.74–1.15)	0.488	27	0.09	0.46 (0.18–1.20)	0.114
Hemorrhagic stroke	Non-PMDs	945	0.13	Ref.		36	0.10	Ref.	
	PMDs	85	0.11	0.80 (0.64–1.00)*	0.048	26	0.09	0.71 (0.34–1.49)	0.362
Ischemic stroke	Non-PMDs	4,649	0.64	Ref.		223	0.63	Ref.	
	PMDs	615	0.78	1.18 (1.08–1.28)*	<0.001	221	0.77	1.27 (1.08–1.50)*	0.003
Other cerebrovascular disease	Non-PMDs	3,024	0.41	Ref.		159	0.45	Ref.	
	PMDs	358	0.45	1.06 (0.95–1.18)	0.292	131	0.45	1.00 (0.82–1.23)	0.968
Emboli and thrombosis	Non-PMDs	3,815	0.52	Ref.		189	0.54	Ref.	
	PMDs	420	0.53	0.97 (0.88–1.07)	0.515	161	0.56	0.99 (0.81–1.20)	0.893
Heart failure	Non-PMDs	2,212	0.30	Ref.		115	0.33	Ref.	
	PMDs	195	0.25	0.78 (0.68–0.90)*	<0.001	70	0.24	0.58 (0.41–0.80)*	0.001
Arrhythmia	Non-PMDs	13,827	1.90	Ref.		749	2.14	Ref.	
	PMDs	2,043	2.63	1.31 (1.25–1.37)*	<0.001	722	2.52	1.13 (1.04–1.23)*	0.005
Conduction disorder	Non-PMDs	1,603	0.22	Ref.		80	0.23	Ref.	
	PMDs	214	0.27	1.21 (1.06–1.39)*	0.006	75	0.26	1.19 (0.86–1.64)	0.295
Other type of CVDs	Non-PMDs	9,433	1.29	Ref.		459	1.31	Ref.	
	PMDs	1,110	1.42	1.04 (0.98–1.10)	0.231	405	1.41	1.03 (0.92–1.15)	0.621

We used stratified Cox regression to calculate the HRs and 95% CIs, in which two-tailed Wald tests were used to calculate *P* values. We did not adjust for multiple comparison.

^a^Estimates were adjusted for birth year, county of residence, educational level, country of birth, civil status, income, use of hormonal contraceptives, use of hormone therapy and history of psychiatric disorders.

*P < 0.05.

Educational level and history of psychiatric disorders modified the association between PMDs and CVDs in the population analysis (*P* for interaction < 0.05), but not in the sibling analysis (Extended Data Table 3). The association was only significant in women who did not initiate HRT in both the population and sibling analyses. In addition, the association was not clearly modified by initiation of hormonal contraceptives in both analyses (*P* for interaction > 0.05).

We observed a positive association between PMDs and CVDs in all time intervals in analyses stratified by time since the index date, although we lacked statistical power when analyzing HRs after the 10-year follow-up in the sibling analysis (Extended Data Table 4). In addition, comparable associations were found after additional adjustment for parity, or after additional adjustment for body mass index (BMI), and smoking among parous women (Extended Data Table 5); when additionally adjusting for diabetes, asthma, autoimmune diseases, menstrual disorders, polycystic ovary syndrome (PCOS), dyslipidemia and medication use (Extended Data Table 6); when restricting the analysis to women in counties with available primary care register data (Extended Data Table 7); or when restricting to women who received two consecutive PMD diagnoses that are at least 28 days apart (Extended Data Table 8).

## Discussion

With prospectively collected data, comprehensive adjustment for important confounders (including familial factors) and robust findings in sensitivity analyses, our study illustrated that women who received a PMD diagnosis in specialist or primary care are at a higher risk of CVDs. The risk was particularly pronounced for PMDs diagnosed in earlier ages, as well as PMDs with comorbid PND. In addition, such positive association was significant for several types of CVDs in both the population and sibling analyses, including hypertensive diseases, essential hypertension, ischemic heart disease, cerebrovascular disease, ischemic stroke and arrhythmia.

Small cross-sectional studies have reported an adverse cardiovascular profile among women with PMDs. For example, a study in Nigeria found higher blood pressure in the late luteal phase than in the early luteal phase, only in women with PMDs but not in other women^25^. Other studies have found higher blood pressure^26^ and higher levels of triglycerides^27^ among women with PMDs. However, studies linking PMD to CVD endpoints are scarce and inconclusive. Chung et al. found that women with PMS did not have a higher risk of hypertension^20^, whereas Bertone-Johnson et al. found that women with PMDs had a 40% higher risk of hypertension^19^. In our study, we found that women with PMDs have a 5% higher risk of hypertensive disorders than others, supporting a mild association with hypertension.

We are not aware of studies assessing whether other cardiovascular events are associated with PMDs. In our study, both the population and sibling analyses showed a positive association for ischemic heart disease, cerebrovascular disease, ischemic stroke and arrhythmia. Considering that the median age at end of follow-up was 42 years in the population analysis, further studies with a longer follow-up to incorporate high-risk age groups of CVDs are warranted.

The mechanism underlying the association between PMDs and CVDs may be multifactorial. First, some studies indicated potential shared genetic risk factors between PMDs and CVDs, such as polymorphism in estrogen receptor alpha^21,22^. However, in our study, the association remained materially unchanged in the sibling analysis, suggesting that genetic confounders are unlikely to explain our results.

Alternatively, shared nongenetic risk factors between PMDs and CVDs may explain the association. For example, both PMDs and CVDs are associated with BMI^15,28^ and smoking^14,29^. However, we observed significant associations after adjusting for these factors. In addition, PMDs are highly comorbid with depression and anxiety^30^, which may underlie the association between PMDs and CVDs. However, we observed an association of PMDs with CVDs among individuals without any psychiatric disorder. In addition, studies have shown that some menstrual characteristics, for example, age at menarche, and menstrual disorders such as excessive menstruation, irregular menses and dysmenorrhea are associated with CVD risk^31,32^. Our results minimally changed after adjustment for menstrual disorders, suggesting that the association between PMDs and CVDs is independent of these menstrual variables. Although the confounding effect of age at menarche might have been partially controlled for in the sibling analyses^33^, such information was not available in registers and future studies could further explore such influence.

Furthermore, PMDs are plausibly biologically linked to CVDs. Premenstrual edema symptoms, including breast tenderness and swelling of extremities, are associated with dysregulation of the RAAS^18^, which plays a significant role in vasoconstriction and hypertension^34^. In addition, progesterone may have differential effects on metabolic profiles in women with and without PMDD^35^. Indeed, limited evidence has shown that women with PMDs have a higher level of total cholesterol than others, and premenstrual symptom burden is associated with metabolic syndrome^27,36^. Furthermore, emerging evidence has shown that women with PMDs have higher levels of inflammation^16,17^, which is fundamental in the process of atherogenesis^37^ and subclinical atherosclerosis^38^. Notably, we found a stronger association for women who have developed both PMDs and PND than for those having PMDs only. PMDs and PND are both characterized as abnormal sensitivity to hormone fluctuations^39^. PND has been linked to CVDs in previous studies, and both PMDs and PND entail dysregulated inflammation^40^ and cardiometabolic alterations^41^. However, future studies may further investigate the potential mechanisms.

Our study is a nationwide population-based study with a maximum of 22 years of follow-up, including comprehensive data on disease phenotypes from primary care, specialist care and death registers. The large sample size enabled us to conduct stratified analyses and estimate associations for PMDs with specific types of CVDs, whereas the sibling analyses allowed us to minimize confounding from familial confounders. However, our study has limitations. First, the diagnosis of PMDs has not been validated in the Swedish registers. However, analyses restricted to consecutive PMD diagnosis at least 28 days apart generated similar results. In addition, as women at the index date were not aware of the future status of their CVDs, such misclassification should be non-differential and would only have attenuated our results^42^. Second, there could be potential misclassifications in CVDs as well. For example, hypertensive disorders may be underestimated in regions where we did not have primary care data. However, analyses restricted to women who lived in counties where primary care register data were available yielded similar results. Third, women with PMDs may have more healthcare visits and thus be more likely to have CVDs detected. Nevertheless, we still observed positive associations after 10 years’ follow-up. Fourth, although we adjusted for a comprehensive set of confounders (for example, familial confounders in sibling analyses; smoking and BMI in both analyses), we cannot rule out residual confounding, for example, nutrition intake^43,44^ and alcohol use^45,46^. Finally, PMDs identified in this study may represent a patient group who experience more severe symptoms. Therefore, our findings may not generalize to individuals with milder PMDs.

In conclusion, our study shows that women with PMDs diagnosed in specialist or primary care have a higher risk of CVDs, suggesting that women with PMDs would face health challenges in the long run. Our results need to be replicated in other populations, and healthcare professionals can be aware of the potential CVD risk when managing women with PMDs, particularly those who developed both PMDs and PND. In addition, if the results are confirmed in other populations, PMDs may be considered as a factor for future CVD risk stratification and prediction among women in the real world.

## Methods

### Ethics approval

This study is approved by the Swedish Ethical Review Authority (2020-06540). Informed consent is waived for register-based studies in Sweden. We have complied with all relevant ethical regulations.

### Study design

We conducted a nationwide population-based matched cohort study followed by a sibling comparison. We used multiple Swedish national registers, including the Total Population Register (TPR), National Patient Register (NPR), Causes of Death Register (CDR) and Prescribed Drug Register (PDR). We also used data from regional primary care registers in the regions of Stockholm, Skåne, Uppsala, Västra Götaland and Värmland, which cover 58–62% of women of reproductive age between 2001 and 2022 in Sweden. These registers are cross-linked through a unique personal identification number. Details of the registers are summarized in Extended Data Table 9.

The study base comprised women born between 1949 and 2006, namely, women aged 16 years (>96% of Swedish girls have menarche by 16 years of age^47^) to 52 years (average age at menopause in Sweden^48^) in 2001–2022. We excluded women who emigrated, died, had bilateral oophorectomy or hysterectomy according to the NPR, or had a diagnosis of PMDs, before immigration to Sweden, age 16 years or 1 January 2001, whichever came later, leaving 3,644,105 women eligible for inclusion (Fig. 1).

The NPR records diagnoses made by specialists in all inpatient care and >80% of outpatient care in Sweden, and the primary care register includes diagnoses made by general practitioners. In our study, we had access to data from the primary care registers of five counties in Sweden. We identified PMD diagnoses made by specialists from the NPR or by general practitioners from the primary care register, based on the International Classification of Diseases, Ninth Revision (ICD-9) and Tenth Revision (ICD-10) (Supplementary Table 1). To supplement PMD ascertainment in counties where data from the primary care register were not available, as described previously^49^, we searched the nationwide PDR for serotonin reuptake inhibitors or hormonal contraceptives with a written indication for PMDs. Although currently there is no validation study for the diagnosis of PMDs, gynecological and psychiatric diagnoses in the NPR have been shown with a good validity^50–53^. For example, the positive predictive value (PPV) is 98% for endometriosis^50^ and 88% for depression^51^ in the inpatient diagnoses of the NPR.

We then classified any individual who had a PMD diagnosis in 2001–2022 as exposed and used the date of diagnosis as the index date. For comparison, each exposed woman was individually matched to 10 women without PMDs at the index date, on birth year and county of residence, using incidence density sampling.

To address familial factors that may confound the association between PMDs and CVDs, we also designed a sibling matched cohort^54^. Briefly, based on the Multi-Generation Register, which included information on biological parents in Sweden^55^, we identified full sisters of the exposed women through a link to the common mother and father. The exposed individuals were matched with their sisters who were free of PMDs at the index date, that is, matched by sisterhood.

In both the population and sibling cohorts, women who had a history of CVDs recorded by ICD-9 and ICD-10 before the index date were excluded, leaving 99,411 women with PMDs and 947,263 matched women in the population cohort and 36,061 women with PMDs and 45,451 unaffected sisters in the sibling cohort.

The study was approved by the Swedish Ethics Review Authority (2021-02775). Informed consent is waived for register-based studies in Sweden.

### Ascertainment of CVDs

Using ICD codes (Supplementary Table 1), we identified the first primary or secondary diagnosis of CVDs dated in the NPR, primary care register and CDR (that is, death attributable to CVDs). Moreover, we studied eight major subgroups of CVDs, including hypertensive diseases, ischemic heart disease, cerebrovascular disease, emboli and thrombosis, heart failure, arrhythmia, conduction disorder and other types of CVDs^56,57^. For this analysis, the first diagnosis for each subtype was used regardless of other subtype diagnoses. The validity of CVD diagnoses in these registers is high. For instance, the PPV was 98% for myocardial infarction in the NPR^58^ and 80% for cerebrovascular diseases and ischemic heart disease in the CDR^59^.

### Covariates

Information on covariates was derived at the index date. We retrieved information on demographics, including birth year, country of birth and county of residence from the TPR. We collected information on educational level, civil status and income from the Longitudinal Integration Database for Health Insurance and Labor Market (LISA). A history of psychiatric disorders was identified using ICD codes from the NPR (Supplementary Table 1). Specifically, as PND may share etiologies with PMDs (for example, hormone sensitivity) and has been shown to be associated with CVD risk^39,60^, we also defined PND as having diagnoses of depressive disorders or having antidepressants prescribed from 1 year before pregnancy to 1 year after. We also collected information on use of hormonal contraceptives and HRT at the index date and during follow-up. Information on parity, smoking before pregnancy and BMI at early pregnancy was collected from the Medical Birth Register (MBR) for parous women from the latest pregnancy before the index date. Information on diabetes, asthma, autoimmune diseases, menstrual disorders (including excessive menstruation, irregular menses, dysmenorrhea), PCOS and dyslipidemia before the index date was collected from the NPR and primary care registers (ICD codes can be found in Supplementary Table 1). The number of any unique medications 1 year before the index date was accumulated by identifying prescriptions from the PDR and grouped as 0, 1–2 and ≥3.

### Statistical analysis

All women were followed from the index date until the first diagnosis of CVDs, emigration, death or 31 December 2022, whichever occurred first (Fig. 1). Unexposed women were additionally censored at their first diagnosis of PMDs. We used stratified Cox regression to estimate the HR and 95% CI of CVDs after a PMD diagnosis, by conditioning on the matching set in the population analysis and conditioning on the sibling set in the sibling analysis. Time since the index date was used as the time scale. The proportional hazard assumption was found to hold according to visual inspection of the Schoenfeld residuals.

To illustrate the potential effects of different sets of covariates on the association between PMDs and CVDs, we built three models. We adjusted for birth year and county of residence in model 1, through conditioning on matching set in the population analysis and adjustment in the sibling analysis. This was considered a crude model in the population analysis and a model with adjustment for basic demographics in the sibling analysis. Then, to control for socioeconomic inequities in CVD incidence and potential confounders^61^, in model 2, we additionally adjusted for country of birth, and socioeconomic status, including educational level, civil status and income. In addition, hormone contraceptives or HRT may regulate menstrual cycles and inhibit or promote ovulation^62^ and subsequently influence premenstrual symptoms. Use of HRT and hormonal contraceptives might be associated with CVD risk^63,64^. Therefore, we additionally adjusted for use of hormone contraceptives and HRT, as potential confounders in model 2. In addition, psychiatric disorders are highly comorbid with PMDs^30^ and are associated with a higher risk of CVDs^65^; we additionally adjusted for history of psychiatric disorders in model 3, hypothesizing that the association between PMDs and CVDs may be attenuated to a larger extent. Model 3 was considered the main model because the maximum set of observed confounders were adjusted for.

To assess the potentially differential associations for PMD subtypes with CVDs, we estimated the association for (1) PMDs diagnosed before and after ages 25, 30 and 35 years and (2) PMDs with and without PND. In addition, to provide insights into CVD subtypes, we estimated HRs for CVD subtypes in relation to PMDs.

We performed stratified analyses. First, because individuals with lower socioeconomic status have a higher number of CVD risk factors^61^, we stratified on educational level to examine whether educational level and PMDs may have synergistic interaction in modulating the risk of CVDs. Furthermore, some common pathophysiological pathways have been indicated for both PMDs and CVDs, for example, inflammation^16,66^, which are also involved in many psychiatric disorders^67^; we therefore stratified on history of psychiatric disorders to examine the potential interaction between PMDs and psychiatric disorders. In addition, hormone contraceptive use can be used to treat PMDs, or other premorbid gynecological conditions, which may mitigate CVD risk^63,68^. We therefore also performed a stratified analysis by timing of use of hormone contraceptives, to examine whether hormone contraceptives confounded and mediated the association for PMDs and CVDs. Moreover, as previous studies have found that women with PMDs have a higher risk of early menopause and menopause symptoms^10,12^, and use of HRT may be associated with future CVD risk^64^, we also stratified the analyses by use of HRT, both before and after the index date.

We also performed other additional analyses. First, to examine whether the association between PMDs and CVDs remained robust after an extended period of follow-up, we analyzed by 1, 5 and 10 years since the index date. Second, to minimize residual confounding from parity, smoking and obesity, we additionally adjusted for parity, smoking and BMI—the analysis adjusted for smoking and BMI was performed only among parous women. In addition, diabetes^69,70^, asthma^17,71^, autoimmune diseases^72^, menstrual disorders^31^, PCOS^73^ and dyslipidemia^74^ have been associated with PMDs and/or CVDs. However, increased medication use may indicate worse health status and more healthcare visits, which may bias the observed association. Therefore, to further reduce residual confounding, we additionally adjusted for these variables based on the main model, including diabetes, asthma, autoimmune diseases, menstrual disorders (including excessive menstruation, irregular menses and dysmenorrhea), PCOS, dyslipidemia before the index date and number of unique medications used 1 year before the index date. Third, PMDs and CVDs (especially hypertensive diseases) are commonly attended by primary care; however, our study did not have primary care data for the entire country. We thus estimated the associations of PMDs with risk of hypertensive diseases by restricting to individuals who lived in counties where data from both primary and specialist cares are available at the index date. Finally, although prospective symptom charting is required in PMD diagnosis in Sweden^75^, such information was lacking in registers. Consistent with previous studies^49,54^, we restricted analysis to individuals with two consecutive PMD diagnoses that were at least 28 days apart, presumably leading to a higher validity of the PMD diagnosis.

Data were cleaned using SAS, version 9.4 (SAS Institute) and analyzed using Stata 18.0 (STATA). Two-tailed test with a significance level of 0.05 was used.

### Reporting summary

Further information on research design is available in the Nature Portfolio Reporting Summary linked to this article.

## Supplementary information

Reporting Summary

Supplementary Table 1 International Classification of Diseases and Anatomical Therapeutic Chemical codes used in the present study.

## Figures and Tables

**Extended Data Table 1:**
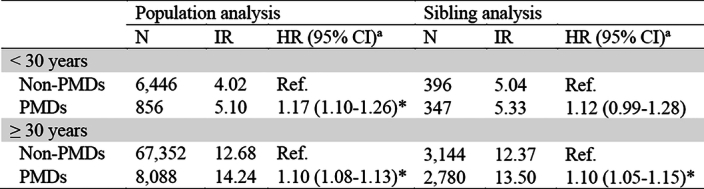
Associations of PMDs with the risk of cardiovascular diseases, stratified by age at baseline (< vs. ≥ 30 years)

CI, confidence interval; CVD, cardiovascular disease; HR hazard ratio; IR incidence rate (per 1000 person-years); N, number of cases; PMD, premenstrual disorder; Ref, reference.

^a^Estimates were adjusted for birth year, county of residence, educational level, country of birth, civil status, income, use of hormonal contraceptives, use of hormone therapy and history of psychiatric disorders.

**Extended Data Table 2:**
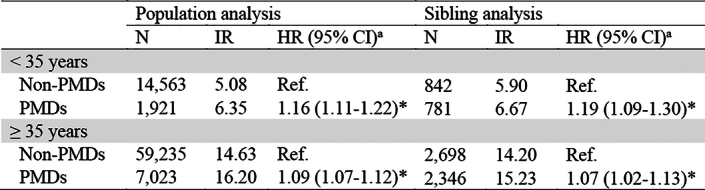
Associations of PMDs with the risk of cardiovascular diseases, stratified by age at baseline (< vs. ≥ 35 years)

CI, confidence interval; CVD, cardiovascular disease; HR hazard ratio; IR incidence rate (per 1000 person-years); N, number of cases; PMD, premenstrual disorder; Ref, reference.

^a^Estimates were adjusted for birth year, county of residence, educational level, country of birth, civil status, income, use of hormonal contraceptives, use of hormone therapy and history of psychiatric disorders.

**Extended Data Table 3:**
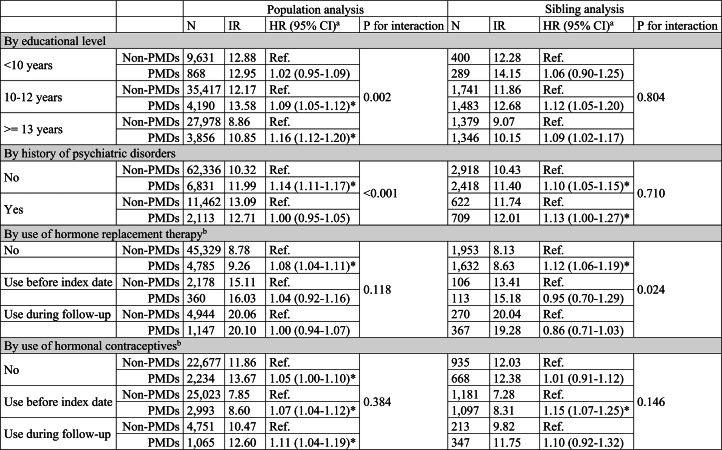
Associations of PMDs with CVDs, stratified by educational level, psychiatric comorbidities, use of hormonal contraceptives, and use of hormone replacement therapy

CI, confidence interval; CVD, cardiovascular disease; HR hazard ratio; IR incidence rate (per 1000 person-years); N, number of cases; PMD, premenstrual disorder; Ref, reference.

^a^Estimates were adjusted for birth year, county of residence, educational level, country of birth, civil status, income, use of hormonal contraceptives, use of hormone therapy and history of psychiatric disorders.

^b^Time-dependent exposure was used. Analysis was restricted to matching sets where the index date was after or at 2006.

*P < 0.05.

**Extended Data Table 4:**
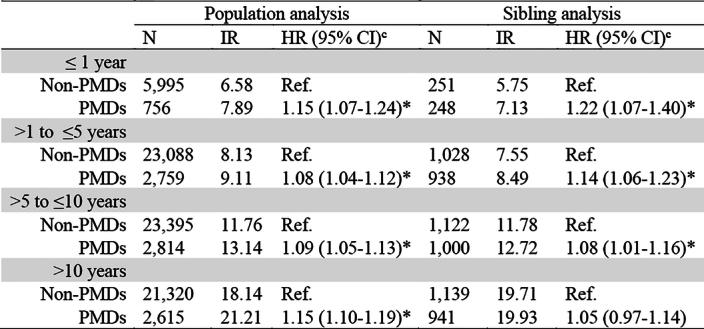
Associations of PMDs with the risk of CVDs, by time since index date

CI, confidence interval; CVD, cardiovascular disease; HR hazard ratio; IR incidence rate (per 1000 person-years); N, number of cases; PMD, premenstrual disorder; Ref, reference.

^a^Estimates were adjusted for birth year, county of residence, educational level, country of birth, civil status, income, use of hormonal contraceptives, use of hormone therapy and history of psychiatric disorders.

*P < 0.05.

**Extended Data Table 5:**
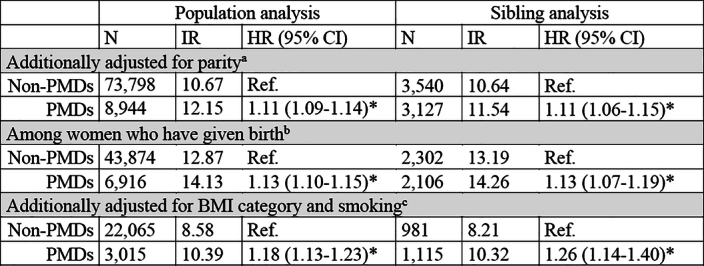
Associations of PMDs with the risk of CVDs, additionally adjust for parity, and additionally adjust for BMI and smoking among women who have given birth in Sweden

BMI, body mass index; CI, confidence interval; CVD, cardiovascular disease; HR hazard ratio; IR incidence rate (per 1000 person-years); N, number of cases; PMD, premenstrual disorder; Ref, reference.

^a^Estimates were adjusted for birth year, county of residence, educational level, country of birth, civil status, income, use of hormonal contraceptives, use of hormone therapy, history of psychiatric disorders and parity.

^b^ Since BMI and smoking were derived in parous women, to improve the comparability with main analysis, we estimated the relationship among parous women. Estimates were adjusted for birth year, county of residence, educational level, country of birth, civil status, income, use of hormonal contraceptives, use of hormone therapy, and history of psychiatric disorders.

^c^Estimates were adjusted for birth year, county of residence, educational level, country of birth, civil status, income, use of hormonal contraceptives, use of hormone therapy, history of psychiatric disorders, BMI category and smoking. Complete-case analysis was used.

*P < 0.05.

**Extended Data Table 6:**
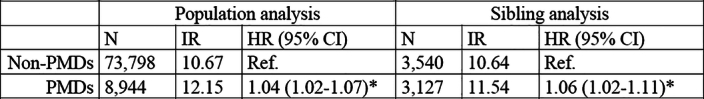
Associations of PMDs with the risk of CVDs, additionally adjusted for diabetes, asthma, autoimmune diseases, menstrual disorders, polycystic ovary syndrome, dyslipidemia, and number of unique medications prescribed before index date

CI, confidence interval; CVD, cardiovascular disease; HR hazard ratio; IR incidence rate (per 1000 person-years); N, number of cases; PMD, premenstrual disorder; Ref, reference.

^a^Estimates were adjusted for birth year, county of residence, educational level, country of birth, civil status, income, use of hormonal contraceptives, use of hormone replacement therapy, history of psychiatric disorders, diabetes, asthma, autoimmune diseases, menstrual disorders, polycystic ovary syndrome, or dyslipidemia before index date, and number of medications prescribed one year before index date (grouped as 0, 1-2, and ≥3).

*P < 0.05.

**Extended Data Table 7:**
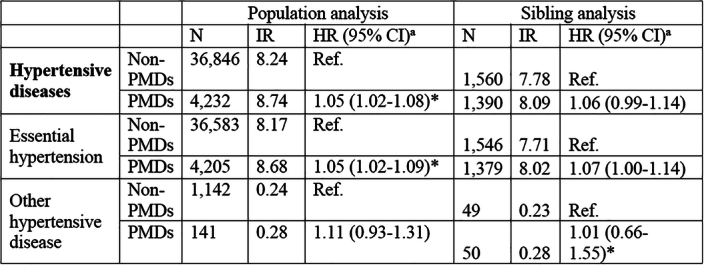
Associations of PMDs with hypertensive diseases, restricting to women who lived in Stockholm, Skåne, Västra Götaland, Uppsala, and Värmland at the index date

CI, confidence interval; CVD, cardiovascular disease; HR hazard ratio; IR incidence rate (per 1000 person-years); N, number of cases; PMD, premenstrual disorder; Ref, reference.

^a^Estimates were adjusted for birth year, county of residence, educational level, country of birth, civil status, income, use of hormonal contraceptives, use of hormone therapy, and history of psychiatric disorders.

*P < 0.05.

**Extended Data Table 8:**
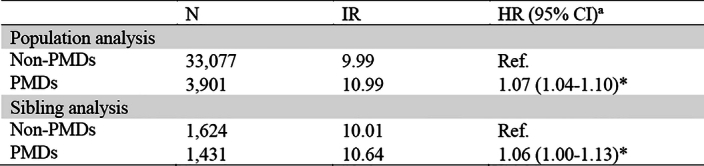
Associations of PMDs with CVDs, restricting to women who receive consecutive PMD diagnoses which are at least 28 days apart

CI, confidence interval; CVD, cardiovascular disease; HR hazard ratio; IR incidence rate (per 1000 person-years); N, number of cases; PMD, premenstrual disorder; Ref, reference.

^a^Estimates were adjusted for birth year, county of residence, educational level, country of birth, civil status, income, use of hormonal contraceptives, use of hormone therapy, and history of psychiatric disorders.

*P < 0.05.

**Extended Data Table 9:**
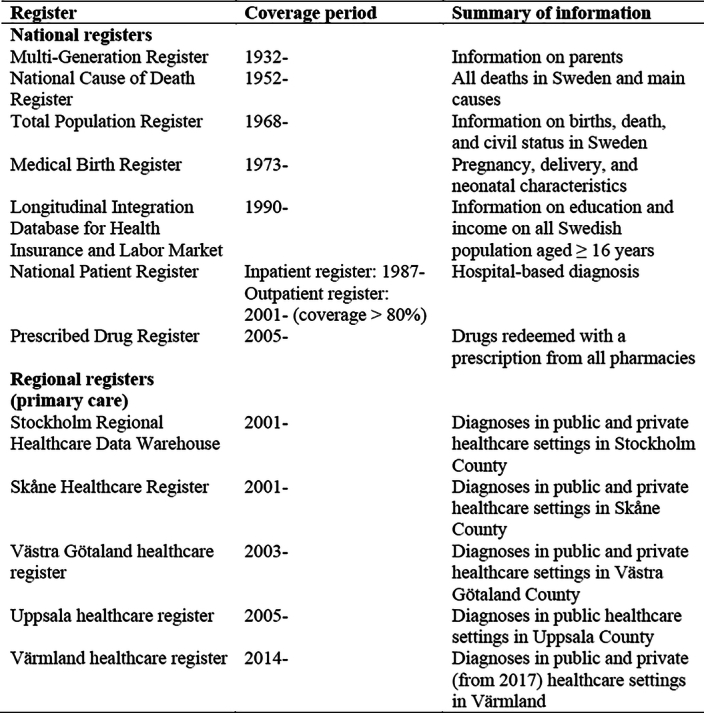
Summary of Swedish registers used in the present study

## Data Availability

The Public Access to Information and Secrecy Act in Sweden prohibits individual-level data from being publicly available. Researchers who are interested in replicating this study can apply for access to individual-level data through Statistics Sweden (https://www.scb.se/en/services/ordering-data-and-statistics/ordering-microdata/). Access to data on patient health can be applied for through Socialstyrelsen (https://www.socialstyrelsen.se/en/statistics-and-data/registers/). Processing of data requests can take 2–4 months.

## Code availability

Analysis coding is available via GitHub at https://github.com/yihuiyang2/PMDCVD.
