# Supplementary material for: Premenstrual disorders and risk of cardiovascular diseases
Source: Nat Cardiovasc Res. 2025 Jul 11;4(8):1001–10. doi: 10.1038/s44161-025-00684-4 (PMC12343293; doi:10.1038/s44161-025-00684-4)
Supplement: Supplementary file 1 — Reporting Summary [file 44161_2025_684_MOESM1_ESM.pdf]

nature portfolio

Corresponding author(s): Yihui Yang and Donghao Lu

Last updated by author(s): May 22, 2025

Reporting Summary

Nature Portfolio wishes to improve the reproducibility of the work that we publish. This form provides structure for consistency and transparency in reporting. For further information on Nature Portfolio policies, see our Editorial Policies and the Editorial Policy Checklist.

Statistics

For all statistical analyses, confirm that the following items are present in the figure legend, table legend, main text, or Methods section.

n/a	Confirmed	
☐	☒	The exact sample size (*n*) for each experimental group/condition, given as a discrete number and unit of measurement
☐	☒	A statement on whether measurements were taken from distinct samples or whether the same sample was measured repeatedly
☐	☒	The statistical test(s) used AND whether they are one- or two-sided<br>*Only common tests should be described solely by name; describe more complex techniques in the Methods section.*
☐	☒	A description of all covariates tested
☐	☒	A description of any assumptions or corrections, such as tests of normality and adjustment for multiple comparisons
☐	☒	A full description of the statistical parameters including central tendency (e.g. means) or other basic estimates (e.g. regression coefficient) AND variation (e.g. standard deviation) or associated estimates of uncertainty (e.g. confidence intervals)
☐	☒	For null hypothesis testing, the test statistic (e.g. *F*, *t*, *r*) with confidence intervals, effect sizes, degrees of freedom and *P* value noted<br>*Give P values as exact values whenever suitable.*
☒	☐	For Bayesian analysis, information on the choice of priors and Markov chain Monte Carlo settings
☒	☐	For hierarchical and complex designs, identification of the appropriate level for tests and full reporting of outcomes
☒	☐	Estimates of effect sizes (e.g. Cohen's *d*, Pearson's *r*), indicating how they were calculated

Our web collection on statistics for biologists contains articles on many of the points above.

Software and code

Policy information about availability of computer code

Data collection	No software was used in data collection.
Data analysis	Data were cleaned using SAS, version 9.4 (SAS institute, Cary, NC) and analyzed using Stata 18.0 (STATA, College Station, TX). Analysis coding can be available at https://github.com/yihuiyang2/PMDCVD.

For manuscripts utilizing custom algorithms or software that are central to the research but not yet described in published literature, software must be made available to editors and reviewers. We strongly encourage code deposition in a community repository (e.g. GitHub). See the Nature Portfolio guidelines for submitting code & software for further information.

Data

Policy information about availability of data

All manuscripts must include a data availability statement. This statement should provide the following information, where applicable:

- Accession codes, unique identifiers, or web links for publicly available datasets
- A description of any restrictions on data availability
- For clinical datasets or third party data, please ensure that the statement adheres to our policy

The Public Access to Information and Secrecy Act in Sweden prohibits individual-level data being publicly available. Researchers who are interested in replicating this study can apply for individual-level data through Statistics Sweden (https://www.scb.se/en/services/ordering-data-and-statistics/ordering-microdata/). Data on patient health can be applied for through Socialstyrelsen (https://www.socialstyrelsen.se/en/statistics-and-data/registers/).

Research involving human participants, their data, or biological material

Policy information about studies with human participants or human data. See also policy information about sex, gender (identity/presentation), and sexual orientation and race, ethnicity and racism.

Reporting on sex and gender	This study studied premenstrual disorders so we only included females. The findings can only apply to females.
Reporting on race, ethnicity, or other socially relevant groupings	We collected information on participants' country of birth from Swedish Total Population Register. We adjusted for it in Model 2 and Model 3, which may help to control the confounding by potentially differential health care-seeking behaviors.
Population characteristics	The median age at index date was 35.4 years in the population analysis and 34.6 in the sibling analysis. Individuals with PMDs were more likely to have a higher educational level, higher parity, and a higher prevalence of psychiatric disorder history at the index date compared to unexposed individuals, in both population and sibling analyses (Table 1). In addition, in the population analysis, individuals with PMDs were more likely to be born in Sweden and use hormonal contraceptives or HRT at the index date than those without PMDs.
Recruitment	The Swedish Total Population Register records nearly all births and migration within Sweden, so the selection bias should be minimized.
Ethics oversight	Swedish Ethics Review Authority

Note that full information on the approval of the study protocol must also be provided in the manuscript.

Field-specific reporting

Please select the one below that is the best fit for your research. If you are not sure, read the appropriate sections before making your selection.

☒ Life sciences ☐ Behavioural & social sciences ☐ Ecological, evolutionary & environmental sciences

For a reference copy of the document with all sections, see nature.com/documents/nr-reporting-summary-flat.pdf

Life sciences study design

All studies must disclose on these points even when the disclosure is negative.

Sample size	No sample size calculation was performed. We determined sample size based on predefined inclusion and exclusion criteria. Specifically, Twe included women born between 1949 and 2006, and excluded those who emigrated, died, had bilateral oophorectomy or hysterectomy, or had a diagnosis of PMDs, before age 16 or January 1, 2001, leaving 3,644,105 women eligible for inclusion. In both population and sibling cohorts, women who had a history of CVDs before the index date were excluded, leaving 99,411 women with PMDs and 947,263 matched women in the population cohort and 36,061 women with PMDs and 45,451 unaffected sisters in the sibling cohort. We have >90% power to detect a 15% increased risk of hypertension in our population.
Data exclusions	We excluded women who emigrated, died, had bilateral oophorectomy or hysterectomy according to the NPR, or had a diagnosis of PMDs, before age 16 or January 1, 2001, leaving 3,644,105 women eligible for inclusion (Figure 1). In both population and sibling cohorts, women who had a history of CVDs before the index date were further excluded. This is because this study aims to assess the association between PMDs and first-onset CVDs, and individuals already diagnosed with CVDs at baseline cannot have their first-onset CVDs during the follow-up. Such exclusion criterion is pre-established.
Replication	We have rerun the analysis coding, which produce the same results as we presented in the manuscript.
Randomization	This is an observational study and no randomization was performed.
Blinding	This is an observational study and no blinding was performed.

Reporting for specific materials, systems and methods

We require information from authors about some types of materials, experimental systems and methods used in many studies. Here, indicate whether each material, system or method listed is relevant to your study. If you are not sure if a list item applies to your research, read the appropriate section before selecting a response.

Materials & experimental systems

n/a	Involved in the study
☒	☐ Antibodies
☒	☐ Eukaryotic cell lines
☒	☐ Palaeontology and archaeology
☒	☐ Animals and other organisms
☒	☐ Clinical data
☒	☐ Dual use research of concern
☒	☐ Plants

Methods

n/a	Involved in the study
☒	☐ ChIP-seq
☒	☐ Flow cytometry
☒	☐ MRI-based neuroimaging

Plants

Seed stocks

Report on the source of all seed stocks or other plant material used. If applicable, state the seed stock centre and catalogue number. If plant specimens were collected from the field, describe the collection location, date and sampling procedures.

Novel plant genotypes

Describe the methods by which all novel plant genotypes were produced. This includes those generated by transgenic approaches, gene editing, chemical/radiation-based mutagenesis and hybridization. For transgenic lines, describe the transformation method, the number of independent lines analyzed and the generation upon which experiments were performed. For gene-edited lines, describe the editor used, the endogenous sequence targeted for editing, the targeting guide RNA sequence (if applicable) and how the editor was applied.

Authentication

Describe any authentication procedures for each seed stock used or novel genotype generated. Describe any experiments used to assess the effect of a mutation and, where applicable, how potential secondary effects (e.g. second site T-DNA insertions, mosiacism, off-target gene editing) were examined.
